# Two versus single needle arthrocentesis of TMJ for internal derangement

**DOI:** 10.6026/973206300210978

**Published:** 2025-05-31

**Authors:** Altaf H. Malik, Irshad A. Misgar

**Affiliations:** 1 Department of Oral Maxillofacial Surgery, Government Dental College, Srinagar, Jammu and Kashmir, India

**Keywords:** TMJ (temporomandibular joint), Internal derangement, arthrocentesis

## Abstract

The efficacy of arthrocentesis with two needles compared with one needle for the treatment of internal derangement of temporomandibular joint (TMJ) is of interest. Fifty patients aged between 17 to 39 years comprised the study material in the department of Oral & Maxillofacial surgery at Govt Dental College Srinagar (India). Clinical evaluation of the patients was done before the procedure and 1 week and 6 months after postoperatively. Intensity of TMJ pain and maximal mouth opening were recorded at each follow-up visit. Arthrocentesis improves the symptoms of the patients with internal derangement with closed lock but addition of other needle didn't improve the results.

## Background:

Internal derangement of Temporomandibular joint disorders (TMDs) though not life threatening are one of the most misdiagnosed and mistreated TMJ disorders affecting about 20-30% of Asian population in the medical practice [1]. Arthocentesis denotes the lavage or flushing of the upper joint compartment by using physiological saline or Hartmann's solution (ringer lactate) using inflow and outflow needles and it is directly derived from TMJ arthroscopy and is in use since 1990; on the basis of the hypothesis that the most effective successful component of TMJ arthroscopy is lavage and is minimally invasive procedure to recapture the disc with elimination of painful mediators by flushing out the painful chemicals [2]. The arthrocentesis resulted in significant improvement in incisal opening and reduction of pain in patients with persistent and severe closed lock as demonstrated by Nitzan [3]. Therefore, it is of interest to assess the efficacy of arthrocentesis with two needles compared with one needle, for the treatment of internal derangement of temporomandibular joint (TMJ).

## Materials and Methods:

This prospective randomized study was conducted on 50 patients who were diagnosed for internal derangement after clinical and MRI correlation: The patients were randomly allocated into two groups. The patients with joint infection, degenerative or autoimmune joint disease, previous joint surgery or arthrocentesis /arthroscopy, were excluded from the study. Only patients with Wilkies stage three or four were included into the study from the study period of year 2022 to 2025. A proper consent was taken from the study after explaining the procedure. Standard auriculotemporal nerve block with 2% lignocaine and local infiltration into the joint was used for anesthesia after patients were properly scrubbed and drapped for the procedure. A Holmund-Helsing line was drawn from mid tragus to the lateral canthus of eye. Two points from mid tragus 10 mm and 2mm down and other point 20mm and 10mm down were labeled as point A and point B respectively. These points corresponded to entry points for 18 or 19 gauge needles in two needle group of 25 patients and in other 25 patients only one needle was used through point A. After insertion of the needle Ringer Lactate was injected into the joint space in open mouth position and jaw was manipulated by opening, protrusive and laterotrusive manipulative movements. A total of 100 ml 0f solution was pushed into joint. In single needle group only point A was used for injection and patients were asked to close the mouth for ejection of solution. The patients were followed for pain, mouth opening, jaw sounds and other post-operative complications or complaints if any. The assessment was done at 1 day, 1 week, 6months and data was entered into master sheet. The pain was measured on VAS scale and mouth opening and lateral movements in millimeters. SPSS 15 was used for data estimation and p value of <0.005 was considered significant.

## Results:

The age of patients ranged from 17 to 39 years (n=50). Mean patient age was 27.96±10.034 years and 36 patients were females. Both the groups were subjected to arthrocentesis, in two needle group the mouth opening improved significantly from 26.14mm to 38.92 mm with p value of < 0.001 (Table 1) with significant drop in pain from 6.92 to 1 (Table 2) on vas scale at 6 monthly time period follow up with p value of <0.001 which indicates significant improvement in symptoms. In single needle group mouth opening increased from 27.17 mm to 38.19 mm with pain vas scores dropping from 6.49 to 0.46 with statically significant difference from pre-operative value, however the intergroup comparison between two needle versus single needle didn't show any statically significant results as p values at follow up ranged always >0.01 at 1 week and 6 months follow up in the intergroup comparison.

## Discussion:

The MRI and clinical pictures vary in co-relation with respect to internal derangement; however there is agreement as regards severity of the displacement and clinical features of internal derangement making it as altered dic and conyle relationship disorder. The stuck disc phenomenon is the result of lot of inflammatory mediaers which leads to alteration of synovial fluid and change of viscosity and negative intra-articular pressure with alteration of mobility of disc or anchoring it to the cranial component of joint [2]. The joint lavage of upper joint space flushes the inflammatory mediators which increase the jaw mobility and the joint adhesions are removed by the flushing action and subsequent mobility of jaw which reduces the pain also. The introduction of needle into joint and subsequent lavage eliminated the negative pressure in the joint which mobilizes the disc by reducing the suction cup effect on disc in internal derangement [3, 4- 5]. The arthocentesis aims at removal or reduction of joint adhesions which improves joint mobility and reduced pain by removing the inflammatory exudates [6, 7- 8]. Our study demonstrated a mean preoperative pain VAS score of 6.92 and mean preoperative MVO was 27.17mm in single needle group significantly improved at every follow-up. At the 6-month postoperative follow-up after arthrocentesis, the mean pain VAS scale was 0.46 with a mean difference of 6.46 from the preoperative value. The MVO was 38.19 mm with a mean difference of 11.02 mm from preoperative opening value in single needle group. The more profound inflammation leads to more severe pain and joint dysfunction and more restriction in mouth opening due to pain; however more profound symptoms lead to more promising results on arthocentesis. The VAS pain level and MVO were interrelated, in our study the more severe the pain the less the mouth opening. The arthroscopic studies studies have elucidated that the inflammatory process are responsible for alteration of retrodiscal and discal changes and the TMJ pain [8, 9, 10, 11, 12-13]. It is an accepted therapeutic and diagnostic tool to correct the dysfunctional state by washing away inflammatory products, promoting joint lubrication [1, 14].

The joints with pronounced inflammatory components and those with a less pronounced inflammatory component influence the arthocentesis outcomes [3, 15 and 16]. The present study compares the effectiveness and tolerability of the two-needle technique in the internal derangement of TMJ with that of a single-needle entry. The single needle adopted one needle for both the saline injection and ejection utilizing the pumping efficacy of joint on the manipulation. On each follow up in both groups there was significant improvement in symptoms but intergroup comparison the findings were similar with p value of .01 making it clear the techniques are similar in output (Table 1, Table 2). It is now established fact that higher intra-articular pressure during saline inflow, helps in breaking down the adhesions and is traumatic than the two-needle entry. A short-term trial has given encouraging results concerning the clinical effectiveness of the single-needle approach [17], but needs further studies. The single-needle technique showed to be equally effective as the classical two-needle technique in improving mouth opening and reducing pain, but it was advantageous in terms of tolerability due to one prick. This may be explained by the fact that the reduced trauma due to the positioning of one needle instead of two needles, the second needle counteracts the higher potentially discomforting, intra-articular pressure exerted by the single needle technique with respect to the inflow-outflow circuit [18, 19]. In postoperative period swelling was encountered in some patients due to extravasation of fluid, but it subsided overnight in all the cases without treatments. Transient facial paralysis was also encountered but resolves of its own [19].

## Conclusion:

Arthrocentesis is simple and it is a less technique sensitive procedure to treat internal derangement in certain closed lock patients. The two needles are as good as single needle techniques to produce desired results as regards the reduction in pain and improvement of mouth opening. However, the single needle technique is less traumatic due to single prick with equal results as of two needle technique which is less discomforting.

## Funding:

Nil

## Figures and Tables

**Table 1:** Mouth opening in single vs double needle

**Clinical parameter**	**Pre-op**	**Post-op 1 wk.**	**Post-op 6 month**	**significance**
Mouth opening in mm (two needle)	26.14	37.68	38.92	P<0.001
Mouth opening in mm(single needle)	27.17	37.64	38.19	P<0.001
P value		>0.01	>0.01	

**Table 2:** Comparison of pain on vas scale of single needle vs double needle arthrocentesis

**Clinical parameter**	**Pre-op**	**1 month**	**6 month**	**comparison**
Pain on VAS(Two needle)	6.92	2.6	1	P<0.001
Pain mean on VAS(Single needle)	6.49	2.3	0.46	P<0.001
P value		>0.01	>0.01	
